# Colon Capsule Endoscopy: Review and Perspectives

**DOI:** 10.1155/2016/9643162

**Published:** 2016-09-06

**Authors:** David Friedel, Rani Modayil, Stavros Stavropoulos

**Affiliations:** Winthrop University Hospital, Mineola, NY, USA

## Abstract

Colon capsule endoscopy utilizing PillCam COLON 2 capsule allows for visualization potentially of the entire colon and is currently approved for patients who cannot withstand the rigors of traditional optical colonoscopy (OC) and associated sedation as well as those that had an OC that was incomplete for technical reasons other than a poor preparation. We will then describe the prior experience and current status of colon capsule endoscopy.

## 1. Introduction

Colorectal cancer (CRC) is the second most prevalent cancer in women and third most common cancer in men globally [1]. In the United States and numerous other nations, optical colonoscopy (OC) is the most utilized modality for CRC screening but several other screening options are available internationally including radiographic and endoscopic modalities that diagnose premalignant or malignant lesions such as flexible sigmoidoscopy, computed tomographic colonography (CTC), and air contrast barium enema and most recently colon capsule endoscopy. There are also stool-based exams which are most sensitive for cancers and high-grade adenomas (fecal occult blood and immunochemical test and fecal DNA). Colon capsule endoscopy is attractive because of its noninvasive nature and potential ability to visualize much of the colon similar to optical colonoscopy. However, its detriments include lesser sensitivity than OC for adenomas and cancers, need for more involved preparation than OC, cost/reimbursement, and inability for therapy and biopsy. These factors and the lack of trained video readers have minimized use in the United States, but there has been moderately extensive use in Europe. It is currently approved for subjects who had incomplete colonoscopy due to technical reasons such as endoscope looping and for subjects with lower gastrointestinal hemorrhage who are less than apt candidates for optical colonoscopy. We will review the current literature concerning colon endoscopy and discuss prospects for the future.

## 2. Technical Aspects

**Figure 1:**
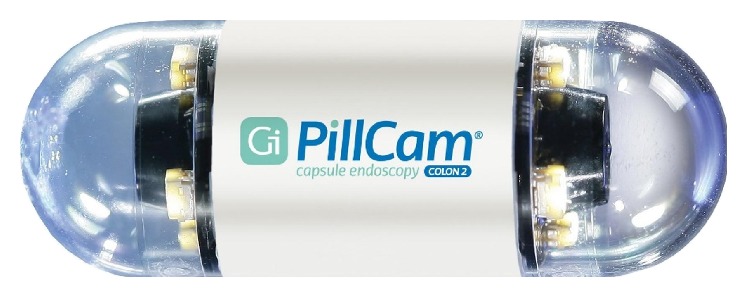
PillCam 2 COLON capsule.

**Figure 2:**
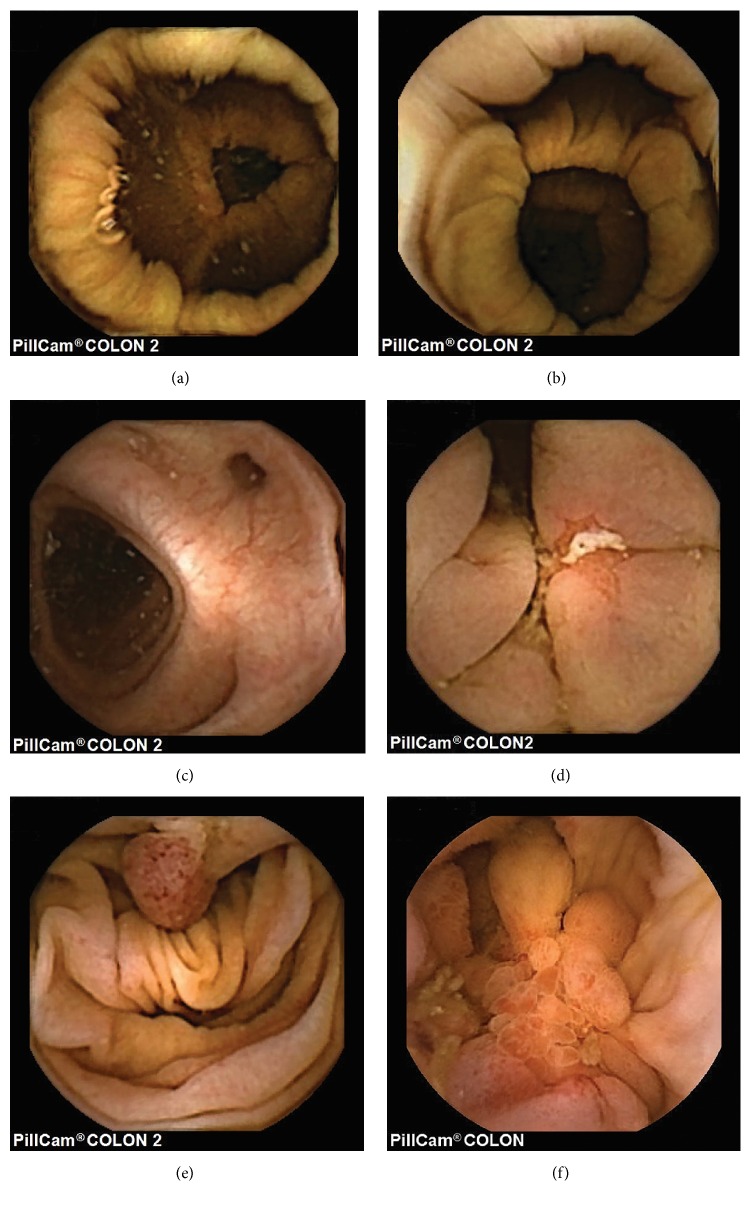
(a) Transverse colon. (b) Ascending colon. (c) Diverticulum. (d) Crohn's disease. (e) Colon polyp. (f) Colon cancer.

Virtually all experience with colon capsule endoscopy has been with the first two generations of the Colon Capsule (Given Imaging Ltd., Yoqneam, Israel) device which is similar to that has been used for small bowel imaging for almost two decades. There are three main components in the Given Imaging diagnostic system: an ingestible capsule (second generation) endoscope, a data recorder, and the RAPID viewing workstation. The second-generation CCE (PCC-2) dimensions are 11.6 × 31.5 mm [2] (Figure 1). It has some similarities to the small intestinal capsule (battery life) and the esophageal capsule (two cameras) but has the unique critical feature of an adaptive frame rate (AFR) (Table 1). The AFR is activated once the capsule is in small bowel (the rate in the esophagus and stomach is 14 images/s) and alternates between 4 images each second when the capsule is stationary and changes to 35 images/second when the capsule is moving [2]. The AFR thus both conserves battery life and allows better viewing when moving. This AFR results from bidirectional communication between the capsule and data recorder. Views are comparable to traditional endoscopy but with better resolution (Figure 2). The visualization system for viewing the video after download at the workstation is similar to the Given small bowel capsule.

Contraindications are similar to the small bowel capsule and include intestinal obstruction, high-grade intestinal structures, gastroparesis and other severe motility issues, and poor colon preparation. The manufacturer discourages use in patients with cardiac electrical devices.

## 3. Colon Preparation

Colon preparation is a particular issue for the use of the colon capsule because the capsule is incapable of maneuvering around or washing away debris. In addition, the 10-hour battery life may not allow pan-colonic visualization. The proportion of subjects passing the capsule rectally is referred to as the excretion rate. Therefore, the various regimens are typically more rigorous than for colonoscopy preparation. The capsule is capable of giving feedback to the patient as to its location via vibrations and display instructions [2]. Depending on the particular protocol, the patient, for instance, would take a prokinetic if the capsule is in the stomach for a prolonged period of time and a booster regimen once the device is mobile when villi are detected [2]. One group employed polyethylene glycol (PEG) as both the initial regimen and the booster with the with a prokinetic (metoclopramide, erythromycin, etc.) given after the initial regimen [10]. The capsule was excreted 86% of the time but good visualization of the entire colon was only noted in 60% [10]. Sodium phosphate solution is commonly used as a booster in Europe but has limited use in the United States because of renal toxicity concerns [11]. One group noted 100% sensitivity for findings seen on subsequent colonoscopy after a prep including three days of clear liquids and combined sodium phosphate, PEG, and bisacodyl [3].

## 4. Validation and Comparative Studies

The design of colon capsule studies to date predominantly consisted of performing a colon capsule exam and then the subjects would get a subsequent optical colonoscopy usually on the same or next day and often utilizing the same preparation. The tandem study design assumed that OC was the gold standard, though occasionally the capsule noted polyps not seen on the initial colonoscopy [12]. The first generation colon capsule had only moderate sensitivity and specificity for colon polyps and was hindered by only fair colon cleansing rates perhaps related to nonstandardized colon preparation. These issues are well demonstrated in a publicized NEJM study with 328 subjects who had known or suspected colonic disease [13]. Only 72% had a good to excellent cleansing and 8% did not excrete the capsule. Sensitivity and specificity for all polyps ≥6 mm were 64% and 84%, respectively. Sensitivity and specificity for advanced adenoma were 73% and 79%, respectively. A major concern was that only 14 out of 19 (74%) cancers were detected by the capsule exam [13]. There was a clear positive correlation of capsule exam sensitivity for polyps and cancer and the degree of cleansing of the colon [13]. A meta-analysis of 837 subjects receiving the first generation colon capsule demonstrated similar sensitivity and specificity for polyps ≥6 mm as the NEJM study, and 16 of 21 cancers were identified (76%) [14].

Fortunately, the second-generation colon capsule introduced in 2009 had much improved detection features including an increased angle of view of 172° (the cameras at each end allow almost 360° viewing), an improved data recorder, and most importantly the feature of the adaptive frame rate (AFR) as mentioned above which would allow optimal visualization when the capsule is moving [15]. Seven series (Table 2) using the CCE-2 consisting of over 1000 subjects demonstrate a remarkably better sensitivity and specificity than the first generation with aggregate sensitivities for polyps ≥6 mm >85% and about 90% for polyps ≥10 mm. Virtually all of the cancers were detected by the capsule. There were few adverse effects usually related to the prep. The largest series [7] enrolled 884 subjects but 689 were included in the analysis and the most common exclusion was inadequate preparation or too rapid colon transit, and thus the excellent sensitivity and specificity may be skewed because of this exclusion. Notably all four cancers were detected by capsule. A meta-analysis of over 2400 subjects approximately equally divided by the CCE-1 and CCE-2 use demonstrated a sensitivity for polyps ≥6 mm of 58% (CCE-1) and 86% (CCE-2), respectively. The CCE-1/CCE-2 sensitivity for polyps ≥10 mm was 54%/87%, respectively [16]. The specificities were about the same for the two versions.

There has been only few studies to date which compared colon capsule with CT colonography [17]. One study design had subjects who had an initial incomplete colonoscopy and then had both CCE-2 and CTC exams. OC was repeated if there were significant findings on either of the two antecedent modalities. CCE had about twice the sensitivity as CTC for polyps ≥6 mm (25 versus 12%). Lesions missed by CTC tended to be small and in the proximal colon [17]. There were no missed cancers in a clinical follow-up of 20 months [17]. A Japanese cohort of 66 patients with a prior history of colon polyps or cancer had both CCE and OC. Per-patient and per-polyp sensitivity was 94% and 87%, respectively. Both these studies suggest that CCE may have a role in detecting small (even flat) polyps in a well prepped colon. In another series, 50 subjects with positive FIT stool test had OC, CCE, and CTC [18]. The sensitivity for CCE and CTC for polyps ≥6 mm was both about 88%, but CCE was preferred by patients over CTC. The sensitivity of CCE for colon lesions and its safety after incomplete colonoscopy has been well validated [19].

## 5. Inflammatory Bowel Disease

Currently, CCE is not supported by evidence as a diagnostic modality for the surveillance of patients with suspected or known inflammatory bowel disease [2, 20]. The diagnosis of ulcerative colitis requires histologic verification. Patients with known UC have accurate assessment of mucosal inflammation by capsule and this may be useful after new drug introduction, alteration, or discontinuation [11, 21]. One study noted that CCE sensitivity and specificity for active colon inflammation in UC were 89% and 75%, respectively [22]. A CCE study in 30 pediatric subjects noted a sensitivity and specificity for disease activity of 96% and 100%, respectively [23]. There is even less literature on CCE use in Crohn's disease and there is a concern for capsule retention due to strictures. There is however significant precedent for capsule use in small intestinal Crohn's disease and some have lauded the colon capsule as a “pan-enteroscopic” test in CD [24].

## 6. Prospects for the Future: Obstacles and Hopeful Developments

One small series of seven subjects touted colon capsule as a primary and sole therapy for presurgery diagnosis of GI malignancy [25], but the evidence to date suggests that the colon capsule is at best a strong ancillary modality to optical colonoscopy in colon cancer screening. Thus, colon capsule will have a similar niche in screening as CTC and stool DNA in that positive findings will prompt a subsequent optical colonoscopy. However, colon capsule will likely to be shown to be superior to the other two modalities in that like optical colonoscopy there will be direct visualization of potential or actual abnormalities and thus likely greater specificity. In addition, colon capsule would be more attractive than CTC because of lack of radiation and need for intravenous access. One analysis demonstrated CC to be much more cost-effective than CTC [26]. Colon capsule like CTC and OC requires a prep and in fact a more prolonged and involved preparation. This together with the relatively prohibitive cost of CC (comparable to OC), current lack of trained video readers (in the US), and lack of biopsy/therapy capabilities are the Achilles heel of colon capsule. Hopefully a preparation regimen that probably does not contain sodium phosphate can be formulated, standardized, and shown to be both effective in cleansing and tolerable. One possibility is a PEG/ascorbic acid combination [27]. There was a dearth of supporting data to include CC as a screening option at the time of the multidisciplinary consortium that generated guidelines colorectal cancer screening in 2008 [28]. Colon capsule is likely to be included in the next set of guidelines for screening.

The reading of the colon capsule typically takes much longer than that for small bowel capsule endoscopy, and the entire gastrointestinal tract is visualized with potential extracolonic findings and pathology. These findings (analogous to CTC) may benefit the patient or alternatively create anxiety and extra testing with incumbent cost. One series of 24 subjects receiving CC noted esophagus/gastric/small intestine pathology in 7/9/14 subjects, respectively [14]. As mentioned, a pan-endoscopic exam would be useful in assessment of Crohn's disease and also drug effect throughout the GI tract.

Technological advances have improved capsule visualization and interpretation. Capsule software allows accurate measurement of a polyp even at a distance [2]. Spectral imaging color enhancement has been incorporated in the Given small bowel capsule and could conceivably be used in the colon version [29]. Computer-based learning of video interpretation and computer-aided analysis have been demonstrated [30, 31].) Experimental prototypes of a capsule steerable in real time and a capsule capable of imaging without a colon prep have been described but are impractical for now [32, 33].

## 7. Conclusion

Colon capsule endoscopy has emerged as a sensitive screening modality for colorectal polyps and cancer ancillary to optical colonoscopy, but the studies to date have not validated it as a primary screening test. It will remain in the armamentarium of ancillary colon screening tests together with CT colonoscopy and stool DNA test. There has been moderately widespread use in Europe, but its use in the United States is limited to relatively few centers, and this is likely to change in the future as there are likely further validating studies, standardization of preparation regimens, lower cost, technological improvements, and more trained readers. It has been used mainly for colon polyp and cancer screening, but it may be proven useful in inflammatory bowel disease and those taking anti-inflammatory drugs. Its noninvasive feature is particularly attractive to patients relative to optical colonoscopy. This may be an option for approximately one-third of all eligible Americans who have not availed themselves of colon screening. In the US, it is predominantly used* for the* approved indications of incomplete colonoscopy due to technical reasons other than inadequate preparation and in those persons who are unable or unwilling to have optical colonoscopy.

## Figures and Tables

**Table 1:** PillCam 2 properties.

Battery life	10 hours
Cameras	2-one at each end
Lenses	3
Angle view	172 degrees
Esophageal-gastric frame rate	14/sec
Small bowel colon	AFR-4/s(stationary) 32–35/s (motion)

**Table 2:** PillCam COLON 2 in colon polyp and cancer detection.

Lead author/*N*/year/reference	Results and comments
Akyuz/28/2016/[3]	Only 28 of 62 subjects in this study focusing on prep had tandem colonoscopy. 6/28 subjects had polyps in 5–10 mm range. Sensitivity, specificity, PPV, and NPV were 100%, 92%, 93%, 100%

Morgan/50/2016/[4]	Of the 50 patients who had tandem capsule and OC 30% and 14% had polyps ≥6 and 10 mm, respectively. For lesions ≥10 mm on OC, capsule sensitivity was 100% and specificity 100%. For polyps ≥6 mm, capsule sensitivity was 93% and specificity 80.0%. The excretion rate was 65%–61% of studies considered adequate cleansing

Hollerhan/62/2014/[5]	62 subjects with (+) FIT had tandem capsule/colonoscopy. Sensitivity/specificity for polyps >6 and 10 mm, respectively, was 95/65% and 89/96%, respectively. 92% had adequate cleaning with 73% excretion rate

Hagel/24/2014/[6]	Sensitivity/specificity for polyps ≥6 mm and 10 mm was 72/91 and 75/100%, respectively. Adequate cleansing 90%. Excretion rate 71%. Extracolonic GI pathology >50%

Rex/884/2013/[7]	Largest series. Sensitivity/specificity for polyps ≥6 and 10 mm was 88/82 and 92/95%, respectively. AC 80%. ER 91%

Spada/117/2015/[8]	8 European centers: 109 subjects analyzed. Sensitivity/specificity for polyps ≥6 and 10 mm was 84/64 and 88/95%, respectively. AC 85%. ER 81%

Eliakim/104/2009/[9]	Initial validating series for CCE-2. Data from 98 subjects. Sensitivity/specificity for polyps ≥6 and 10 mm was 89/76 and 88/89%, respectively. AC 78%. ER 81%
